# Book Review: Principles of Plant-Microbe Interactions: Microbes for Sustainable Agriculture

**DOI:** 10.3389/fpls.2015.00986

**Published:** 2015-11-10

**Authors:** Rama K. Dubey, Vishal Tripathi, P. C. Abhilash

**Affiliations:** Institute of Environment and Sustainable Development, Banaras Hindu UniversityVaranasi, India

**Keywords:** plant-microbe interactions, sustainable agriculture, bioremediaiton

The significance of plant-microbe interactions in sustainable agriculture is enormous. These interactions may be negative such as the host-pathogen interactions leading to the disease development in plants or positive likes the interaction of the plants with the beneficial soil microbiota for stimulating the plant growth, conferring biotic, and abiotic stress tolerance in plants and helping the plants for the revitalization of contaminated and degraded soils (Abhilash et al., 2012). Apart from that, the beneficial microorganisms influence the resource allocation between root and shoot, biodiversity and also mediate the above-ground below ground interactions with herbivores and other natural enemies of the plants. Moreover, such dialogues between plant and microbes can modify the chemical, physical, and biochemical properties of the soil. The root exudate secreted by the plant allocates carbon and nutrients to the soil in the form of low molecular weight sugars, amino acids, and organic acids, polymerized sugars (e.g., mucilage), root border cells and dead root cap cells. Plant secretes phytosiderophores that help in sequestration of metallic micronutrients from the soil. Root exudates also contains secondary metabolites which help in the communication of plant and microbes. However, these interactions are intriguingly complex and dynamic and quite difficult to decipher as they takes places at different interfaces such as rhizosphere, phyllosphere, and endosphere. Therefore, a deep understanding of the interwoven processes taking place at the above interfaces is essential for disentangling the contribution of the each and every player for the ecosystem wellbeing. Thus, it is imperative to understand the key processes of the plant-microbe interactions in relation to assessing the contribution of the plant associated microorganisms to sustainable agriculture, ecosystem restoration, biomass and bioenergy production and mitigating the adverse impacts of climate change (Saleem and Moe, 2014). In this context, the book “*Principles of Plant-Microbe Interactions: Microbes for Sustainable Agriculture*,” edited by Ben Lugtenberg (2015) is a topical and timely contribution on plant-microbe interactions and offers a great hope for harnessing such beneficial interactions for making agriculture as a sustainable enterprise.

Though literature provides ample information on plant-microbe interactions, the current book is first of its kind to discuss not only the interactions of microbes with plants but also the interactions of other important but often ignored players such as nematodes, insects, and pests. The book also discusses various ecological, economic and social aspects related to the plant-microbe based packages right from exploiting the symbiotic relationship to the development of genetically modified organisms for enhancing the sustainability of agriculture. The editor did his level best to incorporating the views of leading scientists and industrial professionals working in the concerned area to give a complete package to students, teachers, academicians, policy makers, young entrepreneurs, biotech, and food industry specialists and policy makers for understanding the plant-microbe interactions and successful exploitation for the benefit of the society. Moreover, the simple and lucid presentation of the crosstalk between microbes and the plants taking place at different interface is good enough to quenches the thirst of the readers from basic to applied and advanced molecular developments in the area.

The editor meticulously divided the book into eight parts for detailing the fundaments of plant-microbe interactions to the application level such as (i) the elucidation of the microbial diversity associated with the plant system and its specific role (ii) the diversity of the phytopathogens, pests mediated crop damage, plant defense, and dilemma about the transgenic crops in public (iii) role of biocontrol agents and transgenics in plant disease resistance and post-harvest loss (vi) mechanism of different plant growth promoting microorganism and arbuscular mycorrhizae in host plant nutrients, water use efficiency and rhizoremediation (v) merit and the challenges in recent techniques like culture independent molecular tools and confocal microscopy for unraveling the rhizosphere microbiome and its interaction with the host plant (vi) the commercialization of microbial inoculum for the plant growth and disease control (vii) harnessing of plant-microbe interactions as a low-input biotechnology and finally (viii) manipulating the plant-microbe interactions for human wellbeing.

The book starts with the fundamentals of the plant-microbe interactions by unraveling the rhizospheric, phyllospheric, and endospheric microbial world associated with the plant system. The book helps in exploring the diverse microbial partners, its importance's and mechanisms of the actions for proper understanding of the topic. It also elucidates the structural and functional details of microbial cell surfaces and its role in exchanging the signals from the exterior to the intracellular milieu. Interestingly, the book also exploring the role of myriad phytopathogens such as bacteria, fungi, nematodes, viruses, and pests its symptomatology, infections mechanisms along with plant immune response to infections and disease control mediated by the biocontrol agents.

Apart from the biocontrol activity, the editor has also made an attempt to address the role of the plant associated microbiome for solubilizing the essential nutrients in soil and also for promoting the plant growth and yields even under adverse environmental conditions. The book also reminds that certain modifications in the microbial traits and rhizosphere environment will enhance the productivity of the agroecosystems. Importantly, the students and restoration workers will get in depth knowledge about various strategies for reshaping the rhizosphere microbiome. Similarly, transferring the genetic machinery of the nitrogen fixation in to non-legume plants also provides new vistas in sustainable agriculture.

The book also unveil the concepts and issues related with the formulation, efficacy testing based on the European Plant Protection Organization, process of registration, and the global commercialization of the microbial inoculums in detail. The major global producers of the microbial inoculums are also detailed in this book. Although microbes like *Pseudomonas* sp*., Bacillus* sp., and *Trichoderma* sp. are the most suitable bio-inoculants, there are always outstanding concerns regarding the shell-life and field performance of these inoculums. Interestingly, the Editor has paid attention to detail the next generation ideal bioinoculants with the concepts of the enhanced stability, carrier suitability, spore forming capability, better inoculation strategies, seed–soil inoculation, microbial inoculants consortium application including bacteria, and fungi.

It has been generally postulated that under changing climatic conditions, the increasing atmospheric CO_2_ will have a fertilization effect on plants and will increase the allocation of nutrients in above- and belowground parts. Hence, it is unclear that how such change will go to affect the plant-microbe interactions at ecosystem level (Abhilash and Dubey, 2014). Although such changing conditions will have a significant impact on plant-microbe interactions, the present book does not shed light on this important issue. Similarly, the exploitation of plant-microbe interactions for the clean-up of contaminated soils has been presented in accordance to the clean-up of organic pollutants with little emphasis on heavy metal and mixed pollutants (organic and inorganic) contaminated soil. Furthermore, in a time when next generation sequencing technologies have been completely revolutionized the microbial community analysis, the current book describes the microbial community analysis mainly on the basis of Denaturing Gradient Gel Electrophoresis. Nevertheless, we enjoyed reading this book as the editor tried to cover almost all fundamental and applied aspects of the plant-microbe interactions. As a final word, the book can be described as *a book for all*.

## Author contributions

RD, VT, and PA wrote the review.

### Conflict of interest statement

The authors declare that the research was conducted in the absence of any commercial or financial relationships that could be construed as a potential conflict of interest.
